# Genetic risk factors in patients with deep venous thrombosis, a retrospective case control study on Iranian population

**DOI:** 10.1186/s12959-015-0064-y

**Published:** 2015-11-10

**Authors:** Soudabeh Hosseini, Ebrahim Kalantar, Maryam Sadat Hosseini, Shadi Tabibian, Morteza Shamsizadeh, Akbar Dorgalaleh

**Affiliations:** Department of Hematology and Blood Transfusion, School of Allied Medicine, Iran University of Medical Sciences, Tehran, Iran; Department of Medical Genetics, Faculty of Medical Sciences, Tarbiat Modares University, Tehran, Iran; Department of Hematology and Blood Transfusion, School of Allied Medicine, Iran University of Medical Sciences, Tehran, Iran; Department of Nursing and Midwifery, Shahroud University of Medical Sciences, Shahroud, Iran

**Keywords:** Deep venous thrombosis, Plasminogen activator inhibitor-1, FV Leiden, Prothrombin20210, Methylenetetrahydrofolate reductase

## Abstract

**Background:**

Venous thromboembolism (VTE) could be manifested as deep venous thrombosis (DVT) or pulmonary embolism (PE). DVT is usually the more common manifestation and is usually formation of a thrombus in the deep veins of lower extremities. DVT could occur without known underlying cause (idiopathic thrombosis) which could be a consequence of an inherited underlying risk factor or could be a consequence of provoking events, such as trauma, surgery or acute illness (provoked thrombosis). Our aim in this study was to assess the impact of some previously reported genetic risk factors including, methylenetetrahydrofolate reductase (MTHFR) C677T and A1298C, plasminogen activator inhibitor-1(PAI-1) 4G/5G, prothrombin 20210 and FV Leiden on occurrence of DVT in a population of Iranian patients.

**Methods:**

This long-term study was conducted on 182 patients with DVT and also 250 age and sex matched healthy subjects as control group. The diagnosis of DVT was based on patient’s history, clinical findings, D-dimer test, and confirmed by Doppler ultrasonography. After confirmation of DVT, both groups were assessed for the five mentioned mutations. The relationship between mutations and predisposition to DVT was calculated by using logistic regression and expressed as an OR with a 95 % confidence interval (CI).

**Results:**

Our results revealed that FV Leiden (OR 6.7; 95 % CI = 2.2 to 20.3; P = 0.001), MTHFR C677T (OR 6.0; 95 % CI = 2.2 to 16.4; *P* < 0.001), MTHFR A1298C (OR 8.3; 95 % CI = 4.4 to 15.8; *P* < 0.001), and PAI*-*1 4G/5G (OR 3.8; 95 % CI = 2.1 to 7.2; *P* < 0.001) mutations were all significantly associated with an increased risk of DVT. Prothrombin 20210 was found in none of the patients and controls.

**Conclusion:**

Our findings suggest that genetic risk factors have a contributory role on occurrence of DVT.

## Background

Venous thromboembolism (VTE) which is manifested either as deep venous thrombosis (DVT) or pulmonary embolism (PE) is the third most frequent cardiovascular disorder after myocardial infarction and stroke. DVT is the thrombotic obstruction of deep veins in the lower extremities, and is a significant cause of mortality and morbidity. According to the studies, 1 in 1000 individuals in old age population is complicated by DVT, annually [1–3].

DVT is a multi-factorial disorder which may occur following a combination of some acquired conditions including surgical procedures, hormonal therapy, trauma, cancer, or immobilization condition (hospitalization), and inherited risk factors [1]. Based on the studies, genetic factors are responsible for approximately 60 % DVT cases [4]. Factor V (FV) Leiden which is the most common cause of inherited thrombophilia, predisposes patients to DVT because of resistance to protein C. Prothrombin 20210, another common cause of hereditary thrombophilia, is also a risk factor for DVT [5]. Two common polymorphisms in methylene tetrahydrofolate reductase (MTHFR) gene including C677T and A1298C, lead to decreased enzyme activity and therefore elevation of homocysteine level. Several studies have shown that these two polymorphisms might be associated with DVT due to hyperhomocysteinemia [6–9]. Another polymorphism which is known as a risk factor for DVT is Plasminogen activator inhibitor-1 (PAI-1) 4G/5G. The 4G allele is associated with a higher level of PAI-1 in plasma which in turn results in a decreased fibrinolysis activity and therefore a higher tendency to thrombus formation [8–10].

There are several studies in the literature which assessed the genetic risk factors of DVT in different ethnical populations [11–15]. In this regard the aim of the current study was to analyze some previously reported genetic risk factors including, MTHFR C677T and A1298C, PAI-1 4G/5G, Prothrombin 20210 and FV Leiden on occurrence of DVT in Iranian population.

## Patients and methods

### Study population, and data collection

This long-term retrospective study was conducted from June 2009 to July 2013 on 182 patients with DVT and also 250 age and sex matched healthy individuals as control group who referred to Clinical laboratory. The mean age of patients was 38.3 ± 15.4 years (median = 34, range = 81), 67.6 % of patients were female and 32.4 % were male. We included patients with confirmed DVT who experienced thrombosis more than once with no specific reason. The exclusion criteria were defined as incomplete medical history. For control group we randomly selected 250 healthy individuals with no history of thrombosis who were as similar as possible to the patients group regarding age and sex (using independent *t*-test, P = 0.4). The mean age of controls was 39.7 ± 16.4 years (median = 36, range = 87), 65.5 % of patients were female and 34.5 % were male. A written consent was obtained from all the participants and the study was approved by the medical ethics committee of local ethical commitee.

Initially each suspected patient was physically examined by physician and also was interviewed by a trained staff about their medical history. The diagnosis of venous thromboembolic disease was based on patient’s history, clinical findings and D-dimer test. Finally DVT was confirmed by Doppler ultrasonography. In addition, all participants were asked to complete a standardized questionnaire on acquired risk factors of venous thrombosis.

### DNA analysis

Blood samples were collected early in the morning in EDTA anticoagulant. Collected whole blood samples were stored at −20 °C for DNA extraction in an appropriate time. In the next step DNA was extracted from thawed whole bloods using QIAAMP DNA blood kit (Qiagen, Germany). The quality and quantity of the extracted DNA was determined by spectrophotometer and also by means of agarose gel electrophoresis. Isolated DNA samples were then stored at −70 °C for long-time usage.

Analysis of PAI-1 polymorphism was performed by amplifying the promoter gene flanking region for 4G/5G polymorphism. The PCR products of 148-bp DNA fragments were digested with *Bse* RI (MBI Fermentas) according to the protocol provided by the manufacturer. Digestion products were subjected to electrophoresis on 2 % agarose gel. The presence of 110- and 38-bp DNA fragments indicated the 4G allele, while the 148-bp fragment indicated the 5G allele. Patients were also screened for Factor V Leiden, prothrombin 20210, MTHFRA1298C and MTHFR C677T gene mutations by procedures which described elsewhere [16–19].

### Statistical methods

Results were reported as mean ± standard deviation (SD) for quantitative variables and percentages for categorical variables. All the statistical analyses were performed by SPSS software. The relationship between the PAI-1, MTHFR and FV Leiden gene polymorphisms and DVT was calculated using logistic regression and expressed as an odd ratio (OR) with a 95 % confidence interval (CI). The combined effect of these polymorphisms on the risk of DVT was also assessed by logistic regression. For this purpose we analyzed the risk of DVT in combined inheritance of each two polymorphisms, either in heterozygous or homozygous manner, in comparison with the single inheritance of the related mutations (as a reference). The *P* values less than 0.05 were considered as statistically significant.

## Results

### Characteristics of the study population

The youngest patient was a 1 year’s female and the oldest one was also an 82 year-old female. Demographic data of patients with DVT is shown in Table 1.

**Table 1:** Demographic data of patients with DVT

	FVLeiden		MTHFR C677T		MTHFR 1298		PAI-1	
	Homo (AA)	Hetero (GA)	Homo (TT)	Hetero (CT)	Homo (CC)	Hetero (AC)	Homo (4G/4G)	Hetero (4G/5G)
Sex								
Male	1 (12.5 %)	5 (50 %)	2 (11.8 %)	3 (100 %)	0 (0)	16 (29 %)	1 (25 %)	5 (14.7 %)
Female	7 (87.5 %)	5 (50 %)	15 (88.2 %)	0 (0)	2 (100 %)	39 (71 %)	3 (75 %)	29 (85.3 %)
Age (year)								
(mean ± SD)	40.3 ± 12.9	42.1 ± 11.2	36.3 ± 13.4	53.3 ± 9.7	25 ± 2.8	31.3 ± 13.1	34.5 ± 16	34.5 ± 11.6
Median	36	44.5	33	51	25	32	28.5	32
Range	35	31	52	19	4	69	35	55

### Prothrombin 20210, Factor V Leiden, MTHFR and PAI-1 mutations analysis

Distribution of FV Leiden, MTHFRC677T, MTHFRA1298C, and PAI-1 4G/5G among DVT patients and controls are indicated in Table 2. Molecular analysis of study population revealed that in the patients group, 8 (4.4 %) patients (1 male and 7 female) were homozygous for FV Leiden and 10 (5.5 %) (5 males and 5 females) were carriers for this mutation. Prevalence of FV Leiden was 9.9 % in patients and 1.6 % in controls. Although none of the controls was homozygous for FV Leiden. Statistical analysis revealed a significant association between FV Leiden mutation and DVT (OR 6.7; 95 % CI = 2.2 to 20.3; P = 0.001). Out of 182 patients 17 (9.3 %) (2 males and 15 females) were homozygous for MTHFRC677T and 3 (1.6 %) were carriers. Among controls 5 were detected with MTHFRC677T mutation, 3 in heterozygous and 2 in homozygous state. The prevalence of this mutation was 10.9 % in patients and 2 % in healthy controls, and therefore the association between MTHFRC677T and DVT were statistically significant (OR 6.0; 95 % CI = 2.2 to 16.4; *P* < 0.001). Among the studied mutations it seems that MTHFRA1298C has the highest prevalence in patients with DVT (31.3 %) and the majority of patients with this mutation were heterozygote (30.2 %). The association between MTHFRA1298C and DVT were also significant (OR 8.3; 95 % CI = 4.4 to 15.8; *P* < 0.001). PAI-1 mutation was found in 38 patients with a prevalence of 20.9 %. Thirty four (18.7 %) out of 38 patients were carriers and only 4 (2.2 %) were homozygous. This mutation was also found in 16 (6.4 %) healthy individuals in heterozygous pattern. A significant association was also found between PAI-1 4G/5G and the occurrence of DVT (OR 3.8; 95 % CI = 2.1 to 7.2; *P* < 0.001). However prothrombin 20210 was found in none of the patients and healthy individuals.

**Table 2:** Distribution of assessed polymorphisms among DVT patients and controls

	FV leiden			MTHFRC677T			MTHFR A1298C			PAI-1		
	Non mutant	Hetero	Homo	Non mutant	Hetero	Homo	Non mutant	Hetero	Homo	Non mutant	Hetero	Homo
Patients with DVT (n = 182)	164 (90.1 %)	10 (5.5 %)	8 (4.4 %)	162 (89.0 %)	3 (1.6)	17 (9.3)	125 (68.7 %)	55 (30.2 %)	2 (1.1 %)	144 (79.1 %)	34 (18.7 %)	4 (2.2 %)
Control group (n = 250)	246 (98.4 %)	4 (1.6 %)	0 (0)	245 (98 %)	3 (1.2 %)	2 (0.8 %)	237 (94.8 %)	9 (3.6 %)	4 (1.6 %)	234 (93.6 %)	16 (6.4 %)	0 (0 %)
OR	6.7			6.0			8.3			3.8		
95 % CI	2.2 to 20.3			2.2 to 16.4			4.4 to 15.8			2.1 to 7.2		
*P*. value	0.001			<0.001			<0.001			<0.001		

We also assessed the combined effect of the four polymorphisms on the risk of DVT; But we found no statistically significant difference (*P* > 0.05 for all the 6 possible combination, including FV Leiden/ PAI-1, FV Leiden/ MTHFR C677T, FV Leiden/ MTHFR A1298C, PAI-1/ MTHFR C677T, PAI-1/ MTHFR A1298C, and MTHFR C677T/ MTHFR A1298C).

## Discussion

Inherited thrombophilia gene mutations are known to have a contributory role in the occurrence of VTE which is clinically manifested by DVT and/or PE [1]. According to the literature the incidence of VTE varies in different races. There have been several studies with the intention of investigating genetic risk factors contributing to DVT in different populations. Akhter et al. indicated a higher prevalence of 4G allele in patients experiencing DVT in Indian Asian population [10]. A similar result was also reported by Shammaa et al. in Lebanese population [20]. In a study on Croatian population by Alfirevic et.al FV Leiden was reported as the highest and prothrombin 20210 as the second most frequent thrombophilia gene mutations [11]. In this regard, we designed this study to investigate the association of five important polymorphisms with the occurrence of DVT in Iranian patients. The incidence of DVT in our studied population was approximately 2 fold higher in females than in males, a finding consistent with the study of Janbabai et al. on northern Iran which noted that DVT is more prevalent in females [21]. According to our results FV Leiden, MTHFR C677T, MTHFR A1298C, and PAI*-*1 4G/5G mutations were all significantly associated with an increased risk of DVT; but prothrombin 20210 was found in none of the patients and controls. These findings are in agreement with the study of Rahimi et al. who showed that FV Leiden but not Prothrombin 20210 is a risk factor for DVT in western Iran [22]. In contrast, there are some reports which failed to find the contributory role of FV Leiden on occurrence of DVT in Iranian patients [23, 24]. In the study conducted by Hajsadeghi et al. FV Leiden and Prothrombin 20210 were introduced as uncommon genetic causes of DVT in Iranian population from Esfahan city. They found no significant association between these two polymorphisms and DVT [23]. These results were supported by the study of Pourgheysari et al. on VTE in central Iran [24]. In a Meta analysis study by Simone et al. involving 11,000 cases and 21,000 controls FV Leiden and Prothrombin 20210 were indicated as moderate risk factors of VTE and the risk was increased in heterozygote carriers of FV Leiden or Prothrombin 20210, however they found no significant association with DVT and homozygous pattern of MTHFR C677T [25]. These contradicting data may reflect the different incidence of various thrombophilia polymorphisms in different populations. Based on studies in different parts of Iran it seems that Prothrombin 20210 is not a significant genetic factor for DVT in Iranian population and there seems to be no consistent findings on FV Leiden.

There are also studies suggesting that combined inheritance of different thrombophilia mutations has a significant effect on the occurrence of DVT [26, 27], while we did not find statistically significant difference between co inheritance of each two polymorphisms and the single inheritance of the related mutations.

A significant limitation of our study was the lack of data regarding clinical characteristics of the studied population such as localization of DVT and acquired risk factors. Considering that DVT is a multi-factorial disorder arising from acquired and genetic risk factors, it is better to assess both clinical and genetic conditions in DVT patients which lead to a more comprehensive and reliable conception of its etiology.

## Conclusion

Our findings suggest that genetic risk factors have a contributory role on occurrence of DVT. But still large studies are required to determine the most important underlying prothrombophilic gene mutations which lead to DVT in Iranian population.

## Abbreviations

VTE: Venous thromboembolism
DVT: Deep venous thrombosis
PE: Pulmonary embolism
MTHFR: Methylenetetrahydrofolate reductase
PAI-1: Plasminogen activator inhibitor-1
CI: Confidence interval
FV: Factor V
